# Effects of a high-prebiotic diet versus probiotic supplements versus synbiotics on adult mental health: The “Gut Feelings” randomised controlled trial

**DOI:** 10.3389/fnins.2022.1097278

**Published:** 2023-02-06

**Authors:** Tanya M. Freijy, Lachlan Cribb, Georgina Oliver, Najwa-Joelle Metri, Rachelle S. Opie, Felice N. Jacka, Jason A. Hawrelak, Julia J. Rucklidge, Chee H. Ng, Jerome Sarris

**Affiliations:** ^1^Professorial Unit, The Melbourne Clinic, Department of Psychiatry, The University of Melbourne, Melbourne, VIC, Australia; ^2^Faculty of Medicine, Dentistry and Health Sciences, Florey Institute of Neuroscience and Mental Health, University of Melbourne, Melbourne, VIC, Australia; ^3^NICM Health Research Institute, Western Sydney University, Westmead, NSW, Australia; ^4^IPAN, School of Exercise and Nutrition Sciences, Deakin University, Melbourne, VIC, Australia; ^5^School of Medicine, Food and Mood Centre, IMPACT Strategic Research Centre, Deakin University, Melbourne, VIC, Australia; ^6^Centre for Adolescent Health, Murdoch Children’s Research Institute, Melbourne, VIC, Australia; ^7^College of Public Health, Medical and Veterinary Sciences, James Cook University, Townsville, OLD, Australia; ^8^School of Pharmacy and Pharmacology, University of Tasmania, Hobart, TAS, Australia; ^9^Human Nutrition and Functional Medicine Department, University of Western States, Portland, OR, United States; ^10^School of Psychology, Speech and Hearing, University of Canterbury, Christchurch, New Zealand

**Keywords:** prebiotics, probiotics, synbiotics, diet, gut microbiota, mood, clinical trial, mental health

## Abstract

**Background:**

Preliminary evidence supports the use of dietary interventions and gut microbiota-targeted interventions such as probiotic or prebiotic supplementation for improving mental health. We report on the first randomised controlled trial (RCT) to examine the effects of a high-prebiotic dietary intervention and probiotic supplements on mental health.

**Methods:**

“Gut Feelings” was an 8-week, 2 × 2 factorial RCT of 119 adults with moderate psychological distress and low prebiotic food intake. Treatment arms: (1) probiotic supplement and diet-as-usual (probiotic group); (2) high-prebiotic diet and placebo supplement (prebiotic diet group); (3) probiotic supplement and high-prebiotic diet (synbiotic group); and (4) placebo supplement and diet-as-usual (placebo group). The primary outcome was assessment of total mood disturbance (TMD; Profile of Mood States Short Form) from baseline to 8 weeks. Secondary outcomes included anxiety, depression, stress, sleep, and wellbeing measures.

**Results:**

A modified intention-to-treat analysis using linear mixed effects models revealed that the prebiotic diet reduced TMD relative to placebo at 8 weeks [Cohen’s *d* = −0.60, 95% confidence interval (CI) = −1.18, −0.03; *p* = 0.039]. There was no evidence of symptom improvement from the probiotic (*d* = −0.19, 95% CI = −0.75, 0.38; *p* = 0.51) or synbiotic treatments (*d* = −0.03, 95% CI = −0.59, 0.53; *p* = 0.92). Improved anxiety, stress, and sleep were noted in response to the prebiotic diet while the probiotic tentatively improved wellbeing, relative to placebo. No benefit was found in response to the synbiotic intervention. All treatments were well tolerated with few adverse events.

**Conclusion:**

A high-prebiotic dietary intervention may improve mood, anxiety, stress, and sleep in adults with moderate psychological distress and low prebiotic intake. A synbiotic combination of high-prebiotic diet and probiotic supplement does not appear to have a beneficial effect on mental health outcomes, though further evidence is required. Results are limited by the relatively small sample size.

**Clinical trial registration:**

https://www.anzctr.org.au/Trial/Registration/TrialReview.aspx?id=372753, identifier ACTRN12617000795392.

## 1. Introduction

The human gut microbiota refers to the microbial population living within the gastrointestinal tract, which consists of over 38 trillion bacterial cells (Sender et al., 2016) and a relatively minor amount of archaea, eukaryotes and viruses (Qin et al., 2010). Diversity estimates indicate more than 1,000 possible gut microbial species across the human population, with at least 160 dominant species per individual (Qin et al., 2010). Adult gut microbiota composition is shaped by various factors (Jernberg et al., 2007; Khachatryan et al., 2008; Mariat et al., 2009; Scott et al., 2013), with diet potentially modulating over 50% of variation (Zhang et al., 2010; Oriach et al., 2016). In Western contexts, a loss of gut microbial diversity has occurred over time, which may in part be explained by the reduced consumption of dietary fibre and consequent depletion of fermentable substrates for the gut microbiota (Deehan and Walter, 2016). A lack of fermentable substrates can lead to the loss of certain bacterial species, loss of diversity, and reduced production of beneficial fermentation end-products such as short-chain fatty acids (SCFAs) (Sonnenburg and Sonnenburg, 2014). Despite the relative stability of the gut microbiota during adulthood (Wu et al., 2011), short-term dietary changes have been shown to swiftly alter its composition (David et al., 2014). Dietary intervention may therefore represent an appropriate strategy to modulate gut microbiota composition.

Preclinical and human studies have yielded extensive evidence of a “microbiota-gut-brain axis,” which is a bidirectional channel of communication between the central nervous system and gut microbiota (see review Cryan et al., 2019). In humans, depression appears to be linked with an altered gut microbiota composition (Naseribafrouei et al., 2014; Jiang et al., 2015; Aizawa et al., 2016; Kelly et al., 2016; Zheng et al., 2016; Valles-Colomer et al., 2019), though there is little consistency regarding specific differences between depressed and healthy individuals. This may be in part due to dietary variation, as only one study (Kelly et al., 2016) has measured nutrient intake. Faecal microbiota transplantation (FMT) research has indicated a potential causal role for the gut microbiota in depression. After FMT from depressed human patients to microbiota-depleted rats, depressive and/or anxiety-like behaviours have been observed in the recipient animals, yet not in animals who received FMT from healthy controls (Kelly et al., 2016; Zheng et al., 2016). Other research suggests that the gut bacterial-induced inflammatory response from exposure to lipopolysaccharide (part of the external membrane of Gram-negative bacteria), may be implicated in the pathogenesis of depression in humans (Cani et al., 2007; Maes et al., 2012, 2013). Various mechanisms underlying the gut-brain axis have been proposed, with a focus on the interplay between neural, endocrine and immune systems (Cryan and Dinan, 2012).

Gut microbiota composition can be modulated via probiotics, which are live bacteria that provide health benefits when ingested in adequate amounts (FAO/WHO, 2002; Hill et al., 2014). Probiotics enhance host health by competitively excluding and inhibiting the growth of pathogens, triggering cytokine synthesis, and producing SCFAs (Kaur et al., 2002). Specific probiotics that have a beneficial effect on mental health, including those from the *Lactobacillus* and *Bifidobacterium* genera (Bravo et al., 2011; Messaoudi et al., 2011; Wang et al., 2016), have been termed “psychobiotics” (Dinan et al., 2013). In one study, 4-week supplementation with *Lactobacillus helveticus* (strain R0052) and *Bifidobacterium longum* (strain R0175) led to improvements in depression and somatisation ratings, problem solving skills, and self-blame scores, and reductions in urinary free cortisol (Messaoudi et al., 2011). Concordantly, probiotic-induced improvements in psychological symptoms, predominantly in clinical populations, have been reported in several meta-analyses (McKean et al., 2017; Ng et al., 2018; Goh et al., 2019; Liu et al., 2019; Nikolova et al., 2019; Chao et al., 2020; Zagórska et al., 2020). Other meta-analytic evidence indicates no probiotic effects in clinical or non-clinical subgroups (Reis et al., 2018).

Another method of modulating gut microbiota composition is through the ingestion of prebiotics, which are non-viable substrates that are selectively utilised by host microorganisms and confer a health benefit (Gibson et al., 2017). Prebiotics are partially or completely fermented by microbes in the large intestine, stimulating the growth of certain protective bacteria, especially bifidobacteria and lactobacilli (Cummings et al., 2001). Benefits of prebiotic ingestion include reducing serum endotoxin concentrations and enhancing intestinal integrity, immunity, blood glucose and plasma lipid levels, and absorption of minerals (Iannitti and Palmieri, 2010; Al-Sheraji et al., 2013), as well as the production of SCFAs (Miller and Wolin, 1979). The most established prebiotics are fructans, including fructo-oligosaccharides (FOS) and inulin; and galacto-oligosaccharides (GOS) (Gibson et al., 2017). These compounds are forms of soluble dietary fibre, and are found in varying quantities in fruits, vegetables, grains, legumes, and nuts. As such plant foods are naturally fibre-rich, prebiotic effects may influence the key differences in gut microbiota composition linked with a long-term high-fibre diet (Simpson and Campbell, 2015). Dietary interventions involving an increase in fibre intake and overall diet quality have been reported to reduce depressive symptoms in individuals with clinical depression (Jacka et al., 2017) and elevated depressive symptoms (Francis et al., 2019; Parletta et al., 2019). Preliminary studies using prebiotic supplements to improve mood have yielded some promising findings (Schmidt et al., 2015; Kao et al., 2016; Johnstone et al., 2021), albeit inconclusive overall, likely due to heterogeneity and the limited research base (Desmedt et al., 2019; Liu et al., 2019; Hofmeister et al., 2021).

The “Gut Feelings” trial was designed to extend the existing literature on prebiotic and probiotic supplementation, and test whether consuming a diet rich in high-prebiotic plant foods would improve gut microbiota composition (Tuohy et al., 2012) and, in turn, enhance mental health. Specifically, we developed a high-prebiotic diet and administered it in comparison with a multi-strain probiotic, and a synbiotic combination of both. We were additionally interested in whether synbiotics [the complementary combination of probiotics plus prebiotics (Saulnier et al., 2008; Swanson et al., 2020)] would produce an additive or synergistic effect and, if so, prove more useful than stand-alone probiotics or a high-prebiotic diet. To our knowledge, this was the first study to examine the effects of a high-prebiotic diet on human mental health, addressing the literature gap concerning a lack of whole-dietary interventions targeting brain function and behaviour (Berding et al., 2021b). Our primary aim was to establish whether dietary intake of prebiotics and/or probiotic supplementation would improve mood in adults with symptoms of psychological distress and low prebiotic food intake, relative to placebo. Secondary aims were to measure treatment effects on depression, anxiety, stress, wellbeing, health-related quality of life (fatigue and wellbeing factors), bowel health, and sleep, and whether treatment response was moderated by intervention adherence. We hypothesised that the synbiotic treatment would outperform all others in improving mood, while both prebiotic and probiotic treatments would be superior to placebo, and that this pattern would remain consistent across measures.

## 2. Materials and methods

### 2.1. Study design

This was an 8-week, 2 × 2 factorial, randomised, placebo-controlled, superiority trial of a probiotic supplement and/or high-prebiotic dietary intervention in 119 non-clinical adult participants with moderate psychological distress and low prebiotic intake. The study was conducted in Melbourne, Australia at an inner city setting (The Melbourne Clinic, Richmond). The trial was prospectively registered in May 2017 (ACTRN12617000795392). Recruitment and intervention delivery occurred over a 23-month period from September 2017 to July 2019. Assessments were completed prior to trial commencement (pre-screening, baseline), during participation (week 2, week 4, week 6), at trial completion (week 8), and at follow-up (week 20). The study received ethical approval from The Melbourne Clinic Research Ethics Committee (TMCREC 289). The study protocol was developed in accordance with the SPIRIT guidelines (Chan et al., 2013). Study outcomes have been reported in accordance with the CONSORT 2010 guidelines (Moher et al., 2010).

### 2.2. Participants

#### 2.2.1. Inclusion and exclusion criteria

Eligible participants had moderate levels of psychological distress [K10 (Kessler et al., 2002) score of 16–26], prebiotic fibre intake <3 g/day (ensuring sufficient scope for dietary improvement), were aged 18–65 years, fluent in written and spoken English, and able to consent to study procedures. Exclusion criteria included fermented food or probiotic supplement use in the prior two weeks, body mass index (BMI) greater than 35, pregnancy, and the presence of gastrointestinal conditions such as irritable bowel syndrome, chronic constipation, or fermentable short-chain carbohydrate (FODMAP) sensitivity. Additional exclusion criteria included having a substance use disorder or clinical psychiatric disorder; using psychotropic medications in the prior 4 weeks; using medications with primarily central nervous system activity; using antibiotics, proton pump inhibitors, non-steroidal anti-inflammatory drugs (>3 doses per month); or using immunosuppressive medications. Individuals who met all criteria except exclusionary antibiotic use were able to participate in the study following a 4-week antibiotic washout period.

#### 2.2.2. Recruitment

Community-dwelling adults were recruited via self-referral in response to online study advertisements and posters displayed in the local area. Potential participants were directed to the study website, containing information about the study and a link to the online pre-screening questionnaire, developed using REDCap (Harris et al., 2009). Those who remained eligible after the questionnaire were contacted by phone if they chose to proceed with further eligibility assessment.

### 2.3. Interventions

Treatment arms: (1) probiotic supplement and diet-as-usual (probiotic group); (2) high-prebiotic diet and placebo supplement (prebiotic diet group); (3) probiotic supplement and high-prebiotic diet (synbiotic group); (4) placebo supplement and diet-as-usual (placebo group).

#### 2.3.1. Supplement intervention

Participants were instructed to take capsules twice-daily (one capsule each morning and evening, with food) for eight weeks. The probiotic formulation, provided by BioCeuticals^®^, delivered 12 billion colony forming units (CFU) per capsule, containing: *Bifidobacterium bifidum* (Bb-06): 2 billion CFU; *Bifidobacterium animalis* subsp. *lactis* (HN019): 1 billion CFU; *Bifidobacterium longum* (R0175): 1 billion CFU; *Lactobacillus acidophilus* (La-14): 2 billion CFU; *Lactobacillus helveticus* (R0052): 2 billion CFU; *Lactobacillus casei* (Lc-11): 2 billion CFU; *Lactobacillus plantarum* (Lp-115): 1 billion CFU; *Lactobacillus rhamnosus* (HN001): 1 billion CFU. Participants were instructed to store the product below 25°C to ensure stability. The placebo capsules, also provided by BioCeuticals^®^, contained an inert substance (microcrystalline cellulose) which appeared identical in appearance, taste, and texture to the probiotic product.

#### 2.3.2. Dietary intervention

The high-prebiotic diet developed for the study was adapted from the Monash University Department of Gastroenterology High Fibre, High-Prebiotic Diet.^1^ Participants were required to ingest seven or more serves per day of prebiotic-rich foods, drawn from a variety of food groups (see Supplementary Table 1). Examples of high prebiotic foods include asparagus, garlic, onion, oats, whole wheat, chickpeas, and watermelon. It was intended that intake of dietary prebiotics would meet a minimum of 5 g per day, the level at which supplemental prebiotics confer physiological and behavioural benefits (Taylor and Holscher, 2020). The serves of prebiotic-rich food were to be introduced into the diet gradually, over a period of five days, to minimise the risk of commonly reported prebiotic side effects such as bloating or flatulence (Cummings et al., 2001). The remainder of the diet was subject to the participant’s discretion. The diet-as-usual control involved participants maintaining their habitual diet throughout the 8-week study.

### 2.4. Procedure

Participants were instructed to undertake a 2-week fermented food and probiotic washout immediately before their baseline appointment, in which probiotic supplements and the following fermented foods were excluded: yoghurt, kefir, pickled vegetables, miso, sauerkraut, kimchi (Korean pickled cabbage), and kombucha tea. Heat-treated fermented foods such as pasteurised sauerkraut and miso soup were excluded because “postbiotic” therapeutic effects can be induced by non-viable (dead) probiotic bacteria (Chudzik et al., 2021). During the baseline session at The Melbourne Clinic, participants provided written informed consent and completed demographic, health, medication, and supplement use questionnaires. The Mini International Neuropsychiatric Inventory 6.0 (Sheehan et al., 1998) and McLean Borderline Personality Disorder (Zanarini et al., 2003) instruments were administered to assess and exclude for clinical psychiatric disorders, and Rome-IV Diagnostic Questionnaire for Functional Gastrointestinal Disorders in Adults (Rome-IV) (Palsson et al., 2016) for bowel disorders. Subsequently, all baseline study assessments were completed.

Eligible participants were randomised and given an 8-weeks’ supply of capsules and instructions on their storage and use. Participants were asked to continue excluding fermented foods and any known probiotics during the 8-week trial period, as well as maintain approximately the same lifestyle, i.e., minimal changes to any current physical activity, self-care practices such as meditation, and micronutrient supplementation. Those allocated to the dietary intervention groups watched a 7-minute introductory video on the high-prebiotic diet delivered by an Accredited Practicing Dietitian specifically for the study. Participants in the non-diet groups were shown a video of similar length which detailed the study procedures, and supplement dosage and storage. Both groups received hard-copy slides of the video content. Those in the diet groups were given a list of high-prebiotic foods with serving sizes, and a hamper of shelf-stable high-prebiotic foods including high-fibre boxed cereals, fruit and nut muesli bars, and dried legumes, as a “starter kit.”

After baseline, assessment of the primary outcome was undertaken at each follow-up time point (weeks 2, 4, 6, 8, and 20), while secondary outcomes were reassessed at week 8 only. Safety monitoring was undertaken via a phone call at week 1, and assessments at weeks 2, 4, 6, and 8. Participants attended in-person at baseline, week 2 and week 8, while the remainder of assessments were performed online.

### 2.5. Outcomes

#### 2.5.1. Primary outcome

The primary outcome was total mood disturbance (TMD) score on the Profile of Mood States Adult Short Form, 2nd edition (POMS 2-SF) (Curran et al., 1995). The POMS 2-SF provides a self-report measure of psychological distress experienced over the past week. It consists of 35 items on a 5-point Likert scale (0–4) and assesses six mood subscales. TMD is calculated as the sum of the scores for five negative mood subscales (anger-hostility, confusion-bewilderment, depression-dejection, fatigue-inertia, tension-anxiety), with the remaining positive mood subscale (vigour-activity) subtracted from this total. As higher TMD values indicate negative mood states, a decrease in TMD indicates improvement.

#### 2.5.2. Secondary outcomes

Secondary outcomes included self-report measures of anxiety on the Beck Anxiety Inventory (BAI) (Beck et al., 1988), depression on the Beck Depression Inventory-II (BDI) (Beck et al., 1996), stress on the Perceived Stress Scale (PSS) (Cohen et al., 1983), wellbeing on the WHO-5 Wellbeing Index (WHO-5) (World Health Organizationion Regional Office for Europe, 1998), sleep on the Leeds Sleep Evaluation Questionnaire (LSEQ) (Parrott and Hindmarch, 1978), and the wellbeing and fatigue subscales of the Short Form Survey-36 (SF-36) (Ware and Sherbourne, 1992). Lower scores indicate better outcomes for BAI, BDI, PSS, while lower scores indicate worse outcomes on the SF-36, WHO-5, and LSEQ. Additional outcomes were BMI (weight (kg)/[height (m)^2^]), and bowel health on the Rome-IV (Palsson et al., 2016), [which contains the Bristol Stool Form Scale (Lewis and Heaton, 1997)]. The Rome-IV reporting timeframe was altered to “past-month” for administration at week 8.

Dietary intake was measured by the Monash University Comprehensive Nutrition Assessment Questionnaire^2^ (CNAQ), with reporting timeframe altered to “past month.” The CNAQ is a validated (Barrett and Gibson, 2010), 297-item food frequency questionnaire (FFQ) that assesses nutrient intake, including prebiotic dietary fibres. In addition, a purpose-built online dietary screener, modified from the Dietitians Australia Healthy Eating Assessment (see footnote 2)^3^, was developed to assess eligibility and monitor dietary adherence. The screener measured servings of vegetables, fruit, breads and cereals, dairy, meats and alternatives, discretionary choices, alcohol, prebiotic-rich foods, and fermented foods. A composite dietary quality score (0–100) and estimate of daily prebiotic intake (in grams) were calculated.

#### 2.5.3. Adherence

Supplement adherence was assessed by returned capsule counts at week 8, while dietary adherence was assessed using the purpose-built dietary screener at weeks 0, 2, 4, 6, 8, and 20.

### 2.6. Randomisation and blinding

Permuted block randomisation (four per block) was used to assign participants to one of four interventions in an allocation ratio of 1:1:1:1, according to a concealed random allocation sequence. The sequence was generated using randomisation software^4^ and Microsoft Excel, by a disinterested third party. Supplement bottles (provided by BioCeuticals^®^) appeared identical to one another, aside from sequential numbering on the labels. Participants, investigators, and outcome assessors were blinded to the supplement intervention. The nature of the dietary intervention precluded the blinding of participants and investigators, although data cleaning and preparation for analysis was performed blinded to group allocation.

### 2.7. Sample size

Based on a two-tailed analysis with α = 0.05, β = 0.80, and a critical *F*(3,124) of 2.68, we planned to recruit 128 participants to detect a difference (Cohen’s *f* of 0.15; equivalent to Cohen’s *d* of 0.30 for a two-group comparison) between the placebo and treatment groups at post-test. Note that the contrasts of each treatment group against placebo at week 8 (primary outcome) used only half of the available data and therefore had lower statistical power. This power analysis was therefore anti-conservative for this preliminary RCT. Lastly, the sample size calculation did not account for drop-out, though this was partially addressed through employing an intention-to-treat analysis.

### 2.8. Statistical analysis

An intention-to-treat analysis using linear mixed effects models was used to assess treatment effects on the primary outcome (TMD). Such models account for the correlations within subjects and use all available data, including study non-completers. Non-linear effects of time (study visit) were evaluated by comparing model fit between models containing time as a continuous, categorical, and log transformed continuous variable using the Akaike Information Criterion (AIC). Continuous time produced the best fit and was used throughout. All mixed effects models included participant-level random intercepts.

The primary outcome model included baseline score, time, treatment, and treatment × time as fixed effect terms. Treatment was a four-level categorical variable (placebo, prebiotic diet, probiotics, and synbiotics) with placebo as reference category. For the secondary outcomes, observations were only available at baseline and week 8. These models had post-test score as the outcome and treatment and baseline score as predictors (i.e., no random effects), the preferred method in pre-post study designs (O’Connell et al., 2017). Linear regression models were used for secondary outcome scales total PSS, WHO-5, and LSEQ which were approximately normally distributed. Proportional odds (PO) models were used for ordinally scaled secondary outcomes or outcomes with clear non-normality (for instance, due to floor effects): total BAI, total BDI, SF-36 fatigue and wellbeing subscales, and Rome-IV (Bürkner and Vuorre, 2019). PO models were fit using R package *ordinal* (Christensen, 2015). Thresholds for ordinal outcome variables were estimated as equidistant, symmetric, or flexible according to the model which minimised the AIC.

We additionally fit factorial models to assess for interactions between the prebiotic diet and probiotic supplement (Simon and Freedman, 1997). For the primary outcome, the model included terms: baseline score, prebiotic diet, probiotics, prebiotic diet × probiotics, time, prebiotic diet × time, probiotics × time, and prebiotic diet × probiotics × time as fixed effects. Prebiotic diet and probiotics terms were coded as 0.5 for treatment given and −0.5 for treatment not given, respectively. Time was coded such that the final study visit was given value 0. If the two treatments were additive (i.e., did not interact), the coefficient for the prebiotic diet × probiotics interaction term would be zero. A positive coefficient for this interaction indicates that the two treatments are antagonistic while a negative interaction coefficient indicates the treatments are synergistic.

For each secondary outcome we undertook sensitivity analyses in which missing outcome data at week 8 was multiply imputed (see Supplementary Text 1). Cohen’s *d* effect sizes were calculated using the pooled standard deviation at baseline. As we did not adjust for multiple comparisons, secondary outcomes should be considered exploratory. Mixed effects models were fit using R package nlme. R version 4.0.5 was used for all analyses (R Core Team, 2019).

## 3. Results

**FIGURE 1:**
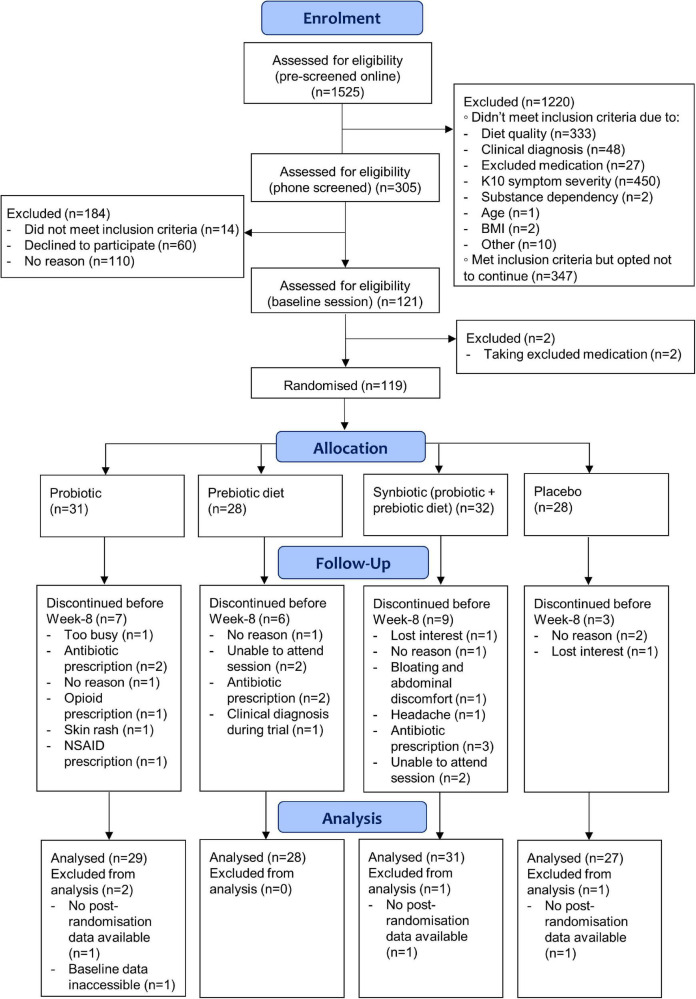
CONSORT flow diagram.

A total of 119 participants were randomised to the four study arms (28 to prebiotic diet, 30 to probiotics, 32 to synbiotics, and 28 to placebo). One participant was not included in the analysis of the primary outcome as a technical error via the MHS online assessment centre led to their baseline POMS 2-SF data being unrecoverable. Therefore, 118 participants were included in the analysis. The sociodemographic and clinical features of the sample are summarised in Table 1. Groups were similar on key demographic and clinical variables. The CONSORT chart describing recruitment and follow-up is displayed in Figure 1. Retention in the trial was moderate with 93 (79%) completing the study. There were six non-completers in the prebiotic diet group, seven in the probiotic group, nine in the synbiotic group, and three in the placebo group, which was similar between groups [χ^2^ (3) = 2.85, *p* = 0.42]. The main reasons for discontinuing were loss of interest/no reason given (*n* = 7), exclusionary medication use (e.g., antibiotics; *n* = 6), inability to attend sessions (*n* = 5), and adverse events (*n* = 3).

**TABLE 1:** Sample socio-demographic and clinical features.

Characteristic	Placebo	Prebiotic diet	Probiotics	Synbiotics
Caucasian, *n* (%)	21 (75.0)	27 (96.4)	25 (80.6)	29 (90.6)
Female sex, *n* (%)	26 (92.9)	27 (96.4)	28 (90.3)	27 (84.4)
**Marital status, *n* (%)**				
Single	12 (42.9)	8 (28.6)	14 (45.2)	13 (40.6)
Married or *de facto*	16 (57.1)	16 (57.2)	17 (54.9)	18 (56.2)
**Education (%)**				
Secondary	1 (3.6)	2 (7.1)	6 (19.4)	2 (6.3)
College or trade certificate	2 (7.1)	4 (14.3)	3 (9.7)	5 (15.5)
Tertiary	25 (89.3)	22 (78.6)	22 (71.0)	25 (48.1)
**Employment (%)**				
Full-time employment	18 (64.3)	12 (42.9)	13 (41.9)	15 (46.9)
Part-time employment	4 (14.3)	6 (21.4)	8 (25.8)	8 (25.0)
Student	6 (21.4)	4 (14.3)	6 (19.4)	3 (9.4)
**Income (%)**				
$0–$39,999	4 (14.3)	4 (14.3)	5 (16.1)	4 (12.5)
$40,000–$79,999	5 (17.8)	5 (17.8)	6 (19.4)	7 (21.9)
$80,000–$99,999	7 (25.0)	6 (21.4)	6 (19.4)	5 (15.6)
$100,000+	12 (42.9)	13 (46.4)	14 (45.2)	16 (50.0)
Age, median (IQR)	32.6 (13.9)	40.1 (16.8)	30.5 (13.7)	38.3 (8.7)
BMI, median (IQR)	25.5 (6.6)	26.1 (4.6)	23.4 (5.2)	24.9 (4.8)

### 3.1. Adherence

Capsule adherence was high in all groups: probiotic 94.1% of total capsules consumed; prebiotic diet 94.4%; synbiotic 94.0%; and placebo 95.8%. Regarding dietary adherence, estimated total prebiotic fibre intake from the purpose-built dietary screener is displayed for dietary and non-dietary groups at each time point in Supplementary Table 2. From weeks 2 to 8, total prebiotic intake was substantially higher in the dietary groups than non-dietary groups (all *p* < 0.001). There were 36 (85.7%) study completers in the dietary groups who increased their intake of prebiotic fibre relative to baseline at all follow-up visits, compared to three (5.9%) in the non-dietary groups.

### 3.2. Assessment of blinding

The success of supplement blinding was assessed via a questionnaire item at week 8, “In your opinion, what formulation did your study capsules contain?” with response options “probiotic,” “placebo,” or “I don’t know.” The responses (Supplementary Table 3) indicate that successful blinding was achieved.

### 3.3. Primary outcome: Mood disturbance

**FIGURE 2:**
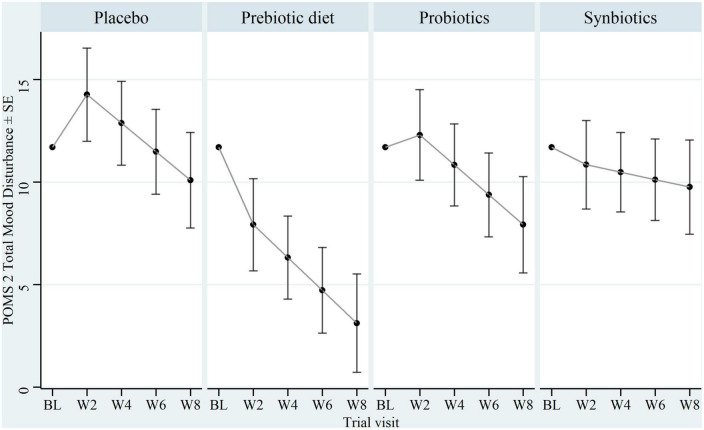
Baseline and estimated follow-up POMS 2-SF total mood disturbance (TMD) score by treatment. Non-factorial model-based estimates of POMS-2 TMD at each visit by group. Model-based predictions are calculated with baseline TMD score at its mean. POMS 2, Profile of Mood States 2-SF; SE, standard error; BL, week 0; W2, week 2; W4, week 4; W6, week 6, W8, week 8.

Results of models comparing each treatment to the placebo group are displayed in Table 2. The effect of treatment on the TMD scale is displayed in Figure 2. At week 8, there was moderate evidence that the prebiotic diet reduced mood disturbance relative to placebo [mean difference (MD) = −6.97; 95% CI = −13.6, −0.345; *p* = 0.039; Figure 1), equivalent to a Cohen’s *d* of −0.60. There was little evidence that the probiotic or synbiotic treatments reduced mood disturbance relative to placebo (MD = −2.17 points; 95% CI = −8.72, 4.38; *p* = 0.51 and MD = −0.331, 95% CI = −6.81, 6.15; *p* = 0.92, respectively). These effect sizes were equivalent to Cohen’s *d* of −0.19 and −0.03, respectively. Notably, there was no difference in the slope of treatment response (rate of change of symptoms) between any of the treatment groups and the placebo group from week 2 to week 8 (treatment × time interaction: *p* = 0.84, *p* = 0.94, and *p* = 0.32 for prebiotic diet, probiotic, or synbiotic group, respectively). That is, each group tended to improve at a similar rate between week 2 and week 8 (see Figure 2). As capsule and dietary adherence rates were both very high, we did not explore whether treatment response was moderated by intervention adherence.

**TABLE 2:** Effect of treatments on primary and secondary outcomes.

Outcome	Treatment	Outcome score, mean (SD), *n*		Mean difference at week 8 (95% CI; *p*)*	Cohen’s *d* (95% CI)
		Baseline	Week-8		
POMS 2-SF TMD	Placebo	11.6 (12.1), 28	10.2 (16.0), 25	Reference	
	Prebiotic diet	10.9 (10.3), 28	3.00 (8.77), 22	−6.97 (-13.6, -0.345; *p* = 0.039)	−0.60 (−1.18, −0.03)
	Probiotic	13.5 (12.3), 30	7.22 (10.3), 23	−2.17 (−8.72, 4.38; *p* = 0.51)	−0.19 (−0.75, 0.38)
	Synbiotic	11.5 (11.7), 32	10.3 (17.2), 23	−0.331 (−6.81, 6.15; *p* = 0.92)	−0.03 (−0.59, 0.53)
PSS total	Placebo	13.5 (5.88), 28	13.0 (7.17), 25	Reference	
	Prebiotic diet	12.5 (4.58), 28	9.50 (4.46), 22	−3.20 (−5.82, −0.575; *p* = 0.017)	−0.61 (−1.11, −0.11)
	Probiotic	12.8 (4.49), 31	10.6 (4.62), 24	−1.65 (−4.22, 0.924; *p* = 0.21)	−0.31 (−0.80, 0.18)
	Synbiotic	12.7 (6.05), 32	10.3 (5.89), 23	−1.89 (−4.49, 0.710; *p* = 0.15)	−0.36 (−0.86, 0.14)
WHO-5 total^1^	Placebo	13.1 (4.57), 27	14.6 (5.10), 25	Reference	
	Prebiotic diet	13.8 (3.80), 28	15.9 (4.99), 22	1.02 (–1.21, 3.25; *p* = 0.37)	0.24 (−0.28, 0.76)
	Probiotic	12.0 (4.51), 31	14.1 (4.25), 24	0.141 (−2.04, 2.32; *p* = 0.90)	0.03 (−0.48, 0.55)
	Synbiotic	13.9 (4.05), 32	14.3 (4.10), 23	−0.498 (−2.70, 1.70; *p* = 0.65)	−0.12 (−0.63, 0.40)
LSEQ average^1^	Placebo	4.85 (1.05), 27	5.08 (1.09), 25	Reference	
	Prebiotic diet	4.67 (0.942), 28	5.74 (1.17), 22	0.770 (0.119, 1.42; *p* = 0.021)	0.73 (0.11, 1.34)
	Probiotic	4.56 (0.899), 31	5.14 (0.94), 24	0.237 (−0.475, 0.879; *p* = 0.47)	0.22 (−0.38, 0.83)
	Synbiotic	4.72 (1.30), 32	5.18 (1.46), 23	0.180 (−0.462, 0.822; *p* = 0.58)	0.17 (−0.44, 0.78)
**Outcome^1^**	**Treatment**	**Outcome score, mean (SD), *n***		OR (95% CI; *p*)*	
		**Baseline**	**Week-8**		
BAI total	Placebo	5.29 (3.10), 28	5.76 (3.89), 25	Reference	
	Prebiotic diet	3.57 (3.17), 28	2.41 (1.99), 22	0.290 (0.102, 0.801; *p* = 0.018)	
	Probiotic	4.87 (3.07), 31	3.29 (2.56), 24	0.394 (0.142, 1.07; *p* = 0.069)	
	Synbiotic	4.00 (3.15), 32	4.04 (4.35), 23	0.480 (0.163, 1.39; *p* = 0.18)	
BDI total	Placebo	5.57 (4.71), 28	4.24 (7.26), 25	Reference	
	Prebiotic diet	5.96 (4.75), 28	2.59 (2.65), 22	0.568 (0.192, 1.65; *p* = 0.30)	
	Probiotic	5.10 (3.88), 31	3.25 (2.92), 24	0.938 (0.343, 2.58; *p* = 0.90)	
	Synbiotic	5.94 (4.57), 32	5.04 (5.80), 23	1.18 (0.410, 3.41; *p* = 0.76)	
SF-36 fatigue^2^	Placebo	49.3 (18.5), 27	53.6 (22.8), 25	Reference	
	Prebiotic diet	50.4 (15.3), 28	59.3 (19.1), 22	1.91 (0.686, 5.41; *p* = 0.22)	
	Probiotic	43.5 (17.2), 31	52.1 (18.5), 24	1.60 (0.602, 4.26; *p* = 0.35)	
	Synbiotic	48.3 (20.3), 32	52.2 (21.2), 23	1.20 (0.436, 3.31; *p* = 0.23)	
SF-36 wellbeing^2^	Placebo	70.8 (13.0), 27	72.6 (14.7), 25	Reference	
	Prebiotic diet	76.9 (10.3), 28	79.3 (8.96), 22	1.61 (0.573, 4.54; *p* = 0.37)	
	Probiotic	69.3 (11.9), 31	77.8 (9.06), 24	2.90 (1.10, 7.77; *p* = 0.032)	
	Synbiotic	71.5 (12.5), 32	74.1 (15.5), 23	1.49 (0.524, 4.23; *p* = 0.46)	

*Model estimates adjusted for baseline outcome score.

^1^Outcomes modelled as ordinal – odds ratios represent fold-change in odds of poorer outcomes (e.g., more severe anxiety) for BAI and BDI scales, and odds of better outcomes for SF-36 fatigue and wellbeing subscales.

^2^Higher values indicate better outcomes. OR, odds ratio; POMS 2-SF, Profile of Mood States 2 – Short Form; BAI, Beck Anxiety Inventory; BDI, Beck Depression Inventory-II; PSS, Perceived Stress Scale; SF-36, Short Form Survey-36.

In the factorial model, there was weak evidence of an antagonistic interaction between the prebiotic diet and probiotic treatment (interaction estimate = 8.80, 95% CI = −0.48, 18.1; *p* = 0.063). This indicated that the change in mood disturbance in the synbiotic group was estimated to be 8.80 points greater, indicating poorer response, than would be expected if the treatments were additive (i.e., no treatment interaction present).

### 3.4. Secondary outcomes

Results for the secondary outcomes are displayed in Table 2. There was moderate evidence that the prebiotic diet reduced symptoms of anxiety on the BAI [odds ratio (OR) for more severe anxiety = 0.290, 95% CI = 0.102, 0.801; *p* = 0.018], perceived stress on the PSS (MD = −3.20, 95% CI = −5.82, −0.575; *p* = 0.017), and improved self-rated sleep on the LSEQ (MD = 0.770, 95% CI = 0.119, 1.42; *p* = 0.021), compared to the placebo group. There was no evidence of benefit on the WHO-5, BDI, and SF-36 fatigue and wellbeing scales (Table 2). For the probiotic treatment, there was moderate evidence of improvement on the SF-36 wellbeing scale (OR for better wellbeing = 2.90, 95% CI = 1.10, 7.77; *p* = 0.032) and weak evidence of improvement in anxiety symptoms (OR for more severe anxiety = 0.394, 95% CI = 0.142, 1.07; *p* = 0.069), compared to placebo. There was no evidence that the synbiotic treatment improved any secondary outcome. No substantive changes to results were found in sensitivity analyses with missing outcome data multiply imputed (Supplementary Table 4). There was no evidence that any treatment influenced BMI at week 8 (all *p* > 0.3).

In factorial models, there was weak evidence of an antagonistic interaction between the prebiotic diet and probiotic supplement on total BAI (*p* interaction = 0.053), total PSS (*p* interaction = 0.12), LSEQ overall average (*p* interaction = 0.078), and SF-36 wellbeing subscale (*p* interaction = 0.12). There was weaker evidence of an antagonistic interaction on WHO-5 (*p* interaction = 0.30), total BDI (*p* interaction = 0.28), and SF-36 fatigue subscale (*p* interaction = 0.21).

### 3.5. Effect of intervention on bowel health symptoms

Changes over time in key Rome-IV bowel health symptoms are plotted for each group in Supplementary Figure 3, with no observable pattern. Symptoms appeared to be mild in general. There was weak evidence that synbiotic treatment increased the odds of having more severe abdominal pain (OR = 3.10, 95% CI = 1.00, 9.99; *p* = 0.053), relative to placebo (Supplementary Table 6). There was no clear evidence that the treatments influenced any other bowel health parameters compared to the placebo group.

### 3.6. Effect of dietary intervention on nutrition outcomes

Total intake of prebiotic oligosaccharides and resistant starch, as well as dietary quality score, are compared between groups who did and did not receive the dietary intervention, in Supplementary Table 5. At week 8, the two diet-treated groups had mean estimated total oligosaccharide intake 3.25 g/day greater than the non-diet groups (95% CI = 2.22, 4.28, *p* < 0.001). Similarly, diet-treated groups had greater mean estimated total resistant starch intake (MD = 2.15 g/day, 95% CI = 1.15, 3.16, *p* < 0.001) and greater dietary quality score (MD = 5.32, 95% CI = 1.57, 9.06, *p* = 0.006).

### 3.7. Week-20 follow-up

Changes in mood disturbance on the POMS 2-SF between week 8 trial completion and week 20 follow-up (*n* = 70) are displayed in Supplementary Figure 1 for each group. At week 20, although mood disturbance was lower in each treatment group compared to placebo, there was little evidence of a difference (MD = −5.85, 95% CI = −13.9, 2.7, *p* = 0.15; MD = −5.07, 95% CI = −13.0, 2.88, *p* = 0.21; MD = −5.97, 95% CI = 14.2, 2.31, *p* = 0.16, for the prebiotic, probiotic, and synbiotic groups, respectively). Supplementary Figure 2 displays average daily prebiotic fibre intake in the diet and non-diet treated groups. At follow-up, prebiotic fibre intake in the diet treated groups dropped substantially to only slightly above baseline levels. There was little evidence that total daily prebiotic fibre intake was higher in diet treated groups than in the non-diet treated groups at follow-up (MD = 0.608 g/day, 95% CI = −0.121, 1.34, *p* = 0.10).

### 3.8. Adverse events

Adverse events were rare and similarly distributed between treatment groups (see Table 3). The most frequently reported adverse events were: bloating, gas, and abdominal discomfort; cold/flu symptoms and sinus issues; changes in bowel movements; and headaches.

**TABLE 3:** Incidence of adverse events across trial by treatment.

Adverse event	Synbiotics	Probiotics	Placebo	Prebiotic diet	Total
Changes in bowel movements	1	1	2	1	5
Back and muscular pain	1	2			3
Bloating, gas, and abdominal discomfort	4		2	1	7
Cold/flu symptoms and sinus issues	3	1		2	6
Fatigue and soreness	1	1	1	1	4
Headaches	1	2	2		5
Bronchitis				1	1
Physical anxiety			1		1
Vomiting, nausea, and dizziness		2	1		3
Swollen lymph nodes				1	1
Weight gain		1			1
Skin rash or fungal infections	1				1
Total (%)	12 (31.6)	10 (26.3)	9 (23.7)	7 (18.4)	38 (100.0)

## 4. Discussion

The Gut Feelings trial assessed the effect of a high-prebiotic diet, probiotic supplement, and their synbiotic combination, on mental health symptoms in adults. We found that the high-prebiotic diet improved symptoms on the primary outcome, TMD, relative to placebo. There were no significant effects for other trial arms. Improvements in anxiety, perceived stress, and sleep were also noted in response to the high-prebiotic diet. For the probiotic supplement, an improvement in wellbeing was noted, although this result should be considered preliminary. Unexpectedly, no benefits were noted in response to the synbiotic combination of probiotics and high-prebiotic diet.

We report that adoption of a high-prebiotic diet for an 8-week duration may induce improvements in global mood disturbance and anxiety. Although there is no previous literature specifically on prebiotic-rich dietary interventions and mental health for comparison, a recent meta-analysis by Liu et al. (2019) investigated the effects of isolated prebiotic fibre supplements and probiotic supplements on psychological symptoms. No significant benefit for symptoms of depression (Cohen’s *d* = −0.08, *p* = 0.51) or anxiety (*d* = 0.12, *p* = 0.11) was found in response to the prebiotic supplements, contrary to our current results for the high-prebiotic diet. Aside from the few studies included in the meta-analysis, there are several potential explanations for this disparity. First, it is possible that isolated preparations of prebiotic fibre could be less effective than a diet high in prebiotic-rich plant foods. Our treatment diet involved consumption of minimally processed high-prebiotic foods across several food groups, promoting intake of a broader range of prebiotic fibres and candidate prebiotics (e.g., short-chain FOS, GOS, inulin, β-glucan, resistant starch, and polyphenols) than most prebiotic supplement formulations, which may induce different fermentation effects (Williams et al., 2017). Second, perhaps the observed mental health benefits from a high-prebiotic diet may be partially attributed to greater vegetable, fruit, and total fibre intake (and consequent changes to dietary macro- and micronutrient composition), as opposed to merely the action of prebiotics (Giri and Sharma, 2022). Indeed, in our study, individuals who followed the high-prebiotic diet not only had significantly higher oligosaccharide and resistant starch intake than non-diet groups at trial completion; they also had greater dietary quality scores than non-diet groups, indicating an overall improvement in their dietary pattern. Further, in natural whole foods, prebiotic fibre occurs in concert with other forms of dietary fibre, minerals, vitamins, phytochemicals, and other bioactive compounds. For instance, oats contain fructans and GOS (Biesiekierski et al., 2011), along with resistant starch, β-glucan, arabinoxylans, sterols, and phenolic compounds (Rose, 2014). Ferulic acid alone, the most commonly found phenolic in whole grains, exhibits anti-inflammatory activity, impacts on redox pathways, and may exert neurological benefits (Neacsu et al., 2017). Given these considerations, it is likely that a high-prebiotic diet facilitates not only gut microbiota-dependent outcomes but also protective effects from dietary improvement in general.

A recent meta-analysis by Firth et al. (2019, 2021) examined studies of dietary intervention on mental health in predominantly non-clinical samples. Relative to inactive control, dietary improvement, which usually includes increasing intake of fibre-rich plant foods, modestly reduced depressive symptoms (Hedge’s *g* = 0.114; *p* = 0.035), but not anxiety symptoms (*g* = 0.077, *p* = 0.302), although there were fewer available data for anxiety. By contrast, in response to our high-prebiotic dietary intervention, we found relatively stronger evidence of benefit to anxiety symptoms than depressive symptoms, as well as reduced TMD. While potentially a chance finding, this divergence may also reflect a distinct biological or psychological effect of a high-prebiotic diet versus interventions targeting dietary quality more broadly; or it might represent a ‘floor effect’ whereby the mild baseline depression symptoms in our sample reduced potential scope for improvement. A third explanation for why a high-prebiotic diet might yield greater mental health symptom improvements than prebiotic supplements is that supplements are easily blinded and placebo controlled, unlike dietary interventions. The greater effect size observed in the current study for the high-prebiotic diet might then be a consequence of placebo effects or biases related to symptom reporting, such as the Hawthorne effect. On the other hand, if such effects were the driving factor in improving endpoints, this should also have been true of the synbiotic intervention. Notably, the high-prebiotic diet used in our study did not alter gastrointestinal symptoms or BMI at trial completion, therefore its possible psychological benefits cannot be attributed to perceived improvements in bowel function, or weight loss, respectively. The latter finding is consistent with another dietary intervention study that targeted individuals with clinical depression, which achieved improvements in depressive symptoms independent of weight changes (Jacka et al., 2017).

Despite the high-prebiotic diet being associated with a reduction in mood symptoms, there was no difference in the slope of treatment response between the prebiotic and placebo groups from weeks 2 to 8, indicating that much of the apparent diet-induced mood benefit occurred during the first two weeks of treatment. The mood benefit was then sustained, but not increased, relative to placebo, at later visits. A similar pattern was noted for the intake of prebiotic fibre in the diet treated groups, which was maintained at a stable level (considerably higher than the non-diet groups) from weeks 2 to 8 (see Supplementary Table 2). As gut microbiota composition and gene expression can be modulated by dietary interventions as brief as 5-days in duration (2014), such a rapid treatment response achieved within the first two weeks of the study may be biologically plausible. As our week-20 follow-up data indicate (Supplementary Figures 1, 2), the mood effect may be reversible, in line with prebiotic intake dropping back down to near-baseline levels, which may be expected due to the gut ecosystem reverting to its previous composition after cessation of the high-prebiotic diet.

During the trial, participants’ substantially higher prebiotic consumption may have stimulated the proliferation of beneficial gut bacteria such as lactobacilli, bifidobacteria, and butyrate-producing species (So et al., 2018), potentially facilitating protective downstream effects on mental health. Mechanistically, such effects may be partially attributed to SCFAs (primarily acetate, butyrate, and propionate), which are gut microbial metabolites produced from prebiotic fermentation. This process may involve both direct fermentation from prebiotics into SCFAs, and indirect fermentation via cross-feeding, in which fermentation end-products of some microbes are utilised by others to produce SCFAs (Holscher, 2017). SCFAs support gut barrier integrity and intestinal immunity, protecting against peripheral inflammation and ultimately, neuroinflammation (Berding et al., 2021a). Butyrate also directly interacts with the central nervous system, regulating the microglial inflammatory response (Huuskonen et al., 2004; Swann et al., 2019). In line with these SCFA anti-inflammatory effects, meta-analytic evidence indicates that oligosaccharide (e.g., FOS or GOS) supplementation reduces C-reactive protein (CRP) concentrations (McLoughlin et al., 2017). SCFAs can also enhance the blood-brain barrier and modulate levels of neurotransmitters and neurotrophic factors (Berding et al., 2021a). Moreover, SCFAs can reduce the cortisol response to psychosocial stress in humans (Dalile et al., 2020), indicating a modulation effect on the HPA axis. Consistent with this, two prebiotic interventions have produced reductions in cortisol levels (Schmidt et al., 2015; Farhangi et al., 2018). Although our study did not measure faecal metabolites or inflammatory markers to trace potential mechanisms, we plan to analyse changes in participants’ faecal microbiota composition and mental health outcomes in a future publication.

In addition to its beneficial effect on the primary outcome, TMD, the high-prebiotic diet also appeared to produce improvements in perceived stress (assessed by PSS) and sleep (LSEQ) at trial completion. The improvement in perceived stress aligns with studies reporting lowered waking cortisol (a stress hormone) after 3-week GOS supplementation (Schmidt et al., 2015), and reduced perceived stress in a within-group, dose-dependent manner after 4-week consumption of a diet high in both prebiotic and fermented foods (Berding et al., 2022). Our results differ from prebiotic interventions that yielded no effects on stress after consumption of FOS (Schmidt et al., 2015), xylo-oligosaccharides (Childs et al., 2014), agave fructans (Ramnani et al., 2015), or inulin-rich vegetables (Hiel et al., 2019). Variation between studies in duration, measures of stress, sample size, and type of prebiotic may partially explain the differing effects. Our finding of an improvement in sleep in response to the prebiotic diet aligns with promising animal (Thompson et al., 2017) and infant (Colombo et al., 2021) research, yet ours appears to be the first human adult prebiotic trial to report a sleep improvement. Human studies that reported no change in sleep used a mixture of FOS and inulin (Smith, 2005; Buigues et al., 2016), or GOS (Johnstone et al., 2021), with varying trial lengths and populations. Interestingly, recent preclinical literature indicates that gut microbial metabolites produced in response to prebiotic fermentation, such as SCFAs or neuroactive steroids, may play a key role in modulating both stress physiology (Thompson et al., 2017, 2020; van de Wouw et al., 2018) and sleep (Thompson et al., 2017). Hence, to clarify effects on stress and sleep, further human research using a high-prebiotic diet is recommended, particularly with faecal metabolome analysis to enable investigation of potential mechanisms.

Although we found that the probiotic treatment produced an improvement in wellbeing (assessed by SF-36 wellbeing subscale) and weak evidence of improved anxiety (BAI), we emphasise that these findings should be interpreted with caution due to their exploratory nature. There was little evidence of benefit for probiotics on the primary outcome, TMD. Recent meta-analyses appear consistent with our probiotic results in similar populations. Liu et al. (2019) found that probiotics produced a reduction in anxiety and depressive symptoms; however, the effect sizes were small (Cohen’s *d* for depression = −0.24; anxiety = −0.10). Moreover, the authors found that larger probiotic-induced mental health effects were evident in clinical versus community samples, with the latter non-significant on their own. Other meta-analyses have reported an absence of probiotic effects on mental health in non-clinical populations (Ng et al., 2018; Reis et al., 2018; Goh et al., 2019; Chao et al., 2020), with one exception (McKean et al., 2017). Considering such information, our findings that probiotics may improve wellbeing or anxiety require further replication in larger samples, and for anxiety, our results align with existing data drawn from non-clinical populations. It should also be noted that probiotic effects appear to be strain-specific (McFarland et al., 2018), therefore a different multi-strain probiotic formulation may produce different results to ours.

An unexpected finding of our study was that the observed mood benefits of a high-prebiotic diet were not apparent when combined with a probiotic supplement. This means that we should be cautious in interpreting our findings as evidence that the prebiotic diet was the driver of the improved outcomes observed in the prebiotic intervention group. In the synbiotic group, there was weak evidence of an antagonistic interaction between prebiotic diet and probiotic supplements for TMD (*p* = 0.063). Specifically, the synbiotic treatment led to an 8.8-point deterioration in POMS 2-SF score compared to the anticipated score had the probiotic and prebiotic treatments been additive, i.e., no interaction present. It is important to note that the antagonistic interaction does not imply that the synbiotic combination of prebiotic diet and probiotics was harmful. Rather, the synbiotic group reached a similar result to the placebo group for TMD at trial completion, which fell far short of the anticipated additive or synergistic effect in a factorial design. Similar weak evidence of antagonistic interactions between prebiotic diet and probiotics were found for secondary outcomes anxiety (assessed via BAI), stress (PSS), sleep (LSEQ), and wellbeing (SF-36 wellbeing subscale). Conclusions regarding the antagonistic interaction, however, should be tempered by the fact that this study was not appropriately powered to detect such an interaction (Simon and Freedman, 1997) and the 95% confidence interval for the interaction effect for the primary outcome was very wide (−0.41 to 18.1).

It is unknown why the synbiotic combination did not appear to result in beneficial effects in line with the prebiotic diet-only intervention, as synbiotics have been reported to stimulate the gut microbiota (Saulnier et al., 2008) and SCFA production (Childs et al., 2014) more than probiotics or prebiotics alone. Further, synbiotics have been shown to improve psychological outcomes (Haghighat et al., 2021; Moludi et al., 2022), reduce inflammatory markers CRP and tumour necrosis factor-α (McLoughlin et al., 2017), decrease oxidative stress (Salehi-Abargouei et al., 2017), and increase levels of serum brain-derived neurotrophic factor (Haghighat et al., 2021). Though random chance cannot be ruled out as an explanation for the differing apparent efficacy of the prebiotic and synbiotic treatments, other explanations may be proposed. First, it may be possible that the ingested probiotic bacteria competed with endogenous microbiota for prebiotic dietary substrates (O’Toole and Cooney, 2008), potentially resulting in less substrate availability for endogenous bacteria such as butyrate-producing species, which may have led to outcomes such as lowered butyrate production. Second, a possible consequence of enhanced stimulation of the gut microbiota via synbiotics may be an increase in bowel symptoms; in such a scenario, treatment benefits may be somewhat diminished by bowel-related discomfort such as bloating. This possibility was investigated in our analysis of the Rome-IV bowel health data. While there was no evidence of worsened bloating, constipation or stool quality, there was suggestive, although inconclusive, evidence that the synbiotic produced an increase in abdominal pain severity relative to placebo (OR = 3.10; *p* = 0.053). The potential interaction of a high-prebiotic diet and probiotic formula should be evaluated in a larger, future trial, and it is recommended that such studies employ a measure of bowel symptoms to elucidate whether symptoms may mediate any mental health changes.

### 4.1. Strengths and limitations

Strengths of our study include the randomisation of the intervention, double blinding of the probiotic intervention, regular assessment of adherence through a purpose-built FFQ, homogeneous baseline mental health, and moderately high participant retention. Weaknesses are additionally recognised. First, participants and investigators were not blinded to dietary intervention allocation, which may have influenced how participants perceived and/or reported symptoms, potentially resulting in bias on self-report measures. Second, the contrast of the prebiotic dietary intervention to the placebo supplement may not represent a truly “placebo-controlled” comparison. Participants may have had greater levels of expectancy about the dietary intervention than the probiotic supplements, and this may not have been adequately “controlled” in the trial. Third, only relatively small sample sizes were available for treatment comparisons. Finally, the external validity of our findings is limited as the sample was composed of non-clinical individuals, most of whom were highly educated and had relatively high socioeconomic status. Additionally, our sample was largely female, and females appear to reap greater mental health benefits from dietary interventions than males (Firth et al., 2019). For these reasons, it is unclear whether our results would extend to males, clinical groups, or populations of different socioeconomic position.

### 4.2. Implications

The results of this study add to the growing body of nutritional psychiatry research (Sarris, 2019), and provide preliminary insight into strategies that may be useful in non-clinical populations. Our findings are somewhat consistent with the previous interventions in depressive illness, where relatively simple dietary changes that included increasing intake of prebiotic-rich whole plant foods were reported to benefit depression (Jacka et al., 2017; Francis et al., 2019; Parletta et al., 2019). Such changes appear to be simple, economical (Opie et al., 2015), cost-effective (Chatterton et al., 2018; Segal et al., 2020), and safe to implement. Moreover, given the anti-inflammatory properties of prebiotics (McLoughlin et al., 2017) and our finding that a high intake of prebiotic-rich foods improves overall diet quality, consuming several serves of prebiotic-rich foods each day on an ongoing basis may also help to reduce the development of physical illnesses often comorbid with mental health problems. Regarding intervention feasibility, although a registered dietitian delivered the dietary education session, it was done via an asynchronous video medium, which enhances potential scalability for larger populations.

## 5. Conclusion

In summary, this was the first RCT to examine the mental health outcomes of a high-prebiotic dietary intervention, probiotic supplements, and their combination. We found that a diet high in prebiotic-rich whole plant foods may reduce self-reported mood disturbance, anxiety, and stress, and improve sleep in non-clinical adults. A high-prebiotic diet might therefore be a useful strategy for promoting mental health in non-clinical populations. We found limited evidence to support the use of the specific probiotic strain combination used in this study, and no evidence for a synbiotic combination of this probiotic with a high-prebiotic diet, for improving mental health in the current sample. Larger confirmatory RCTs in both clinical and non-clinical populations are needed.

## Data availability statement

The datasets presented in this study can be found in online repositories. The names of the repository/repositories and accession number(s) can be found below: https://osf.io/ye8b7/.

## Ethics statement

The studies involving human participants were reviewed and approved by the Melbourne Clinic Research Ethics Committee (TMCREC 289). The patients/participants provided their written informed consent to participate in this study.

## Author contributions

JS, TF, FJ, JH, RO, and JR contributed to conception and design of the study. TF, GO, and JS administered the project. TF and RO developed the intervention. TF and GO delivered the intervention. JS and CN acted as supervisors. TF, LC, and N-JM managed data curation and wrote the original manuscript draft. LC and TF performed the statistical analysis. All authors contributed to manuscript revision and approved the submitted version.

## Footnotes

1
www.monash.edu/medicine/ccs/gastroenterology/prebiotic

2
www.cnaq.com.au

3
www.healthyeatingquiz.com.au

4
www.sealedenvelope.com
