# Supplementary material for: Effects of a high-prebiotic diet versus probiotic supplements versus synbiotics on adult mental health: The “Gut Feelings” randomised controlled trial
Source: Front Neurosci. 2023 Feb 6;16:1097278. doi: 10.3389/fnins.2022.1097278 (PMC9940791; doi:10.3389/fnins.2022.1097278)
Supplement: Supplementary file 1 [file Data_Sheet_1.pdf]

Supplementary Text 1. Supplementary methods: Multiple imputation for secondary outcomes.

For secondary outcome measures, sensitivity analyses were performed in which missing outcome data was imputed using predictive mean matching (50 imputations; R package ‘mice’ [1]). The following variables were used in the model for the imputation of missing data: sex, education, baseline outcome score, treatment, and any adverse events (yes or no).
