# Supplementary material for: Effects of a high-prebiotic diet versus probiotic supplements versus synbiotics on adult mental health: The “Gut Feelings” randomised controlled trial
Source: Front Neurosci. 2023 Feb 6;16:1097278. doi: 10.3389/fnins.2022.1097278 (PMC9940791; doi:10.3389/fnins.2022.1097278)
Supplement: Supplementary file 2 [file Table_1.pdf]

Supplementary Table 1. High-prebiotic diet: List of prebiotic-rich foods, serves per day, and serving sizes

Food group	Serves	Food item*	Serving size**
Vegetables	*2/day*		
		Artichoke, globe head	½ small
		Artichoke, Jerusalem	1 piece, 50 g
		Asparagus	4 spears
		Beetroot	1 baby, 2 rings
		Broccoli	½ cup
		Cabbage, raw	½ cup
		Chicory root, raw	½ cup
		Dandelion greens	½ cup
		Fennel, bulb	½ cup
		Garlic	1 clove
		Kale, raw	1 packed cup
		Parsnip or kohlrabi	½ cup
		Onion, leek, shallot, or spring onion	¼ cup
		Peas, green or snow	½ cup
		Sweetcorn	½ cup kernels, or 1 cob
Legumes	*1/day*		
		Beans, baked	½ cup
		Beans, red kidney or borlotti or lima or haricot, cooked	½ cup
		Beans, mixed, cooked	½ cup
		Beans, soy or edamame, cooked	½ cup
		Chickpeas, including hummus, cooked	½ cup
		Falafel	4 small balls
		Lentil or bean burger patties	½ patty
		Lentils, cooked	½ cup
		Split peas, cooked	½ cup
Fruit	*1/day*		
		Blueberries	½ cup
		Cherries	1/3 cup
		Currant, red	½ cup
		Dried fruit, i.e., figs, dates	2 figs, or 4 dates
		Grapefruit	½ fruit
		Melon: watermelon or honeydew	1 thick slice
		Nectarine	1 med
		Peach, white	1 med
		Persimmon	1 med
		Pomegranate	½ small
		Rambutan	4 pieces
		Tamarillo	2 tamarillos
		Banana	1 small
Grains	*2/day*		
		Biscuit, rye crispbread	1 piece

		Bread, wholewheat or rye	1 slice, ½ roll
		Cereal, All-Bran	½ cup
		Cereal, oats	½ cup dry, 1 cup cooked
		Cereal, wholegrain, e.g., Sustain, flaked barley	1 cup
		Cereal, Weet-bix	1 cup, or 3 biscuits
		Corn thins, plain	2 pieces
		Couscous, cooked	1 cup
		Muesli	1/3 cup
		Muesli bar, plain with dried fruit	1 bar
		Pasta, gnocchi, or wheat noodles, cooked	1 cup
		Wheat berries, barley, or rye, raw	1/3 cup
Nuts	*1/day*		
		Cashews or almonds	20 nuts or 1 Tbsp nut butter
		Pistachios	30 nuts or 1 Tbsp nut butter
		Walnuts	15 nuts or 1 Tbsp nut butter

* Food list adapted from Monash University High Fibre High-Prebiotic Diet, (Biesiekierski et al., 2011), (Muir et al., 2009), and (Muir et al., 2007);
** Serving sizes adapted from CNAQ food frequency questionnaire and Monash University FODMAP Diet mobile application.

References

Biesiekierski, J., Rosella, O., Rose, R., Liels, K., Barrett, J., Shepherd, S., et al. (2011). Quantification of fructans, galacto-oligosacharides and other short-chain carbohydrates in processed grains and cereals. *Journal of Human Nutrition and Dietetics* 24(2)**,** 154-176.

Muir, J.G., Rose, R., Rosella, O., Liels, K., Barrett, J.S., Shepherd, S.J., et al. (2009). Measurement of short-chain carbohydrates in common Australian vegetables and fruits by high-performance liquid chromatography (HPLC). *Journal of Agricultural and Food Chemistry* 57(2)**,** 554-565.

Muir, J.G., Shepherd, S.J., Rosella, O., Rose, R., Barrett, J.S., and Gibson, P.R. (2007). Fructan and free fructose content of common Australian vegetables and fruit. *Journal of Agricultural and Food Chemistry* 55(16)**,** 6619-6627.
