# Supplementary material for: Effects of a high-prebiotic diet versus probiotic supplements versus synbiotics on adult mental health: The “Gut Feelings” randomised controlled trial
Source: Front Neurosci. 2023 Feb 6;16:1097278. doi: 10.3389/fnins.2022.1097278 (PMC9940791; doi:10.3389/fnins.2022.1097278)
Supplement: Supplementary file 3 [file Table_2.pdf]

Supplementary Table 2. Adherence to prebiotic intervention: Estimated prebiotic fibre intake at each visit

Visit	Estimated total prebiotic intake* (g/day), mean (SD)		
	Dietary intervention groups	Non-dietary intervention groups	*P* (between group difference)**
Baseline	2.23 (1.13)	1.98 (0.888)	0.20
Week 2	4.62 (2.09)	1.96 (1.03)	< 0.001
Week 4	4.72 (1.89)	1.92 (0.876)	< 0.001
Week 6	4.87 (2.20)	1.78 (0.815)	< 0.001
Week 8	4.63 (2.51)	1.90 (1.04)	< 0.001
Week 20 (follow-up)	2.56 (1.25)	1.86 (1.74)	0.10

* Dietary prebiotic intake estimates calculated from the purpose-built dietary screener;
** Welch two sample t-test.
