# Supplementary material for: Effects of a high-prebiotic diet versus probiotic supplements versus synbiotics on adult mental health: The “Gut Feelings” randomised controlled trial
Source: Front Neurosci. 2023 Feb 6;16:1097278. doi: 10.3389/fnins.2022.1097278 (PMC9940791; doi:10.3389/fnins.2022.1097278)
Supplement: Supplementary file 4 [file Table_3.pdf]

Supplementary Table 3. Assessment of capsule blinding success at week 8

	Participant's guess, n (%)			
Group	Probiotic	Placebo	Do not know	Total
Probiotic	8 (8.5)	4 (4.3)	12 (12.8)	24 (25.5)
Prebiotic diet*	4 (4.3)	7 (7.4)	11 (11.7)	22 (23.4)
Synbiotic**	7 (7.4)	12 (12.8)	4 (4.3)	23 (24.5)
Placebo	10 (10.6)	9 (9.6)	6 (6.4)	25 (26.6)
Total	29 (30.9)	32 (34.0)	33 (35.1)	94 (100.0)

* Placebo capsule administered;
** Probiotic capsule administered.
