# Supplementary material for: Effects of a high-prebiotic diet versus probiotic supplements versus synbiotics on adult mental health: The “Gut Feelings” randomised controlled trial
Source: Front Neurosci. 2023 Feb 6;16:1097278. doi: 10.3389/fnins.2022.1097278 (PMC9940791; doi:10.3389/fnins.2022.1097278)
Supplement: Supplementary file 5 [file Table_4.pdf]

Supplementary Table 4. Effect of treatments on secondary outcomes with multiply imputed missing week 8 data

Outcome	Treatment	Mean difference at week 8 (95% CI; *p)**
PSS total	Placebo	Reference
	Prebiotic diet	-3.27 (-5.83, -0.712; *p* = 0.013)
	Probiotic	-1.77 (-4.35, 0.797; *p* = 0.17)
	Synbiotic	-1.98 (-4.64, 0.686; *p* = 0.14)
WHO-5 total[2]	Placebo	Reference
	Prebiotic diet	1.05 (-1.09, 3.18; *p* = 0.33)
	Probiotic	0.150 (-2.00, 2.30; *p* = 0.89)
	Synbiotic	-0.500 (-2.62, 1.62; *p* = 0.64)
LSEQ average[2]	Placebo	Reference
	Prebiotic diet	0.771 (0.133, 1.41; *p* = 0.018)
	Probiotic	0.215 (-0.429, 0.859; *p* = 0.51)
	Synbiotic	0.144 (-0.464, 0.752; *p* = 0.64)
Outcome[1]	Treatment	OR (95% CI; *p*)*
BAI total	Placebo	Reference
	Prebiotic diet	0.273 (0.098, 0.763; *p* = 0.014)
	Probiotic	0.387 (0.141, 107; *p* = 0.066)
	Synbiotic	0.538 (0.191, 1.51; *p* = 0.24)
BDI total	Placebo	Reference
	Prebiotic diet	0.525 (0.174, 1.59; *p* = 0.25)
	Probiotic	0.899 (0.310, 2.61; *p* = 0.84)
	Synbiotic	1.20 (0.405, 3.56; *p* = 0.74)
SF-36 fatigue[2]	Placebo	Reference
	Prebiotic diet	1.91 (0.699, 5.73; *p* = 0.19)
	Probiotic	1.53 (0.572, 4.11; *p* = 0.39)
	Synbiotic	1.20 (0.442, 3.29; *p* = 0.71)
SF-36 wellbeing[2]	Placebo	Reference
	Prebiotic diet	1.79 (0.645, 4.99; *p* = 0.26)
	Probiotic	3.27 (1.22, 8.79; *p* = 0.019)
	Synbiotic	1.25 (0.438, 3.55; *p* = 0.68)

* Model estimates adjusted for baseline outcome score;
[1] Outcomes modelled as ordinal – odds ratios represent fold-change in odds of poorer outcomes (e.g., more severe anxiety) for BAI and BDI scales, and odds of better outcomes for SF-36 fatigue and wellbeing subscales; [2] Higher values indicate better outcomes.
OR = odds ratio; POMS 2-SF = Profile of Mood States 2 – Short Form; BAI = Beck Anxiety Inventory; BDI = Beck Depression Inventory-II; PSS = Perceived Stress Scale; SF-36 = Short Form 36.
