# Supplementary material for: Effects of a high-prebiotic diet versus probiotic supplements versus synbiotics on adult mental health: The “Gut Feelings” randomised controlled trial
Source: Front Neurosci. 2023 Feb 6;16:1097278. doi: 10.3389/fnins.2022.1097278 (PMC9940791; doi:10.3389/fnins.2022.1097278)
Supplement: Supplementary file 6 [file Table_5.pdf]

Supplementary Table 5. Prebiotic fibre intake and dietary quality at baseline and week 8.

Dietary outcome	Baseline, mean (SD), n		Week 8, mean (SD), n		Mean difference at week 8 (95% CI; *p*)[1]
	Diet-treated groups	Non-diet groups	Diet-treated groups	Non-diet groups	
Total oligosaccharides, g/day*	6.46 (4.26), 60	5.65 (2.97), 59	8.62 (4.24), 45	4.54 (1.85), 49	3.25 (2.22, 4.28; $p < 0.001$)
Total resistant starch, g/day*	5.05 (3.91), 60	4.02 (2.11), 59	6.83 (4.74), 45	3.59 (1.76), 49	2.15 (1.15, 3.16; $p < 0.001$)
Dietary quality score**	66.5 (10.9), 60	63.8 (12.3), 58	70.9 (9.52), 45	62.9 (12.6), 49	5.32 (1.57, 9.06; $p = 0.006$)

* Calculated from the Monash Comprehensive Nutrition Assessment Questionnaire;
** Calculated from the purpose-built dietary screener;
[1] ANCOVA model estimates adjusted for baseline value.
