# Supplementary material for: Effects of a high-prebiotic diet versus probiotic supplements versus synbiotics on adult mental health: The “Gut Feelings” randomised controlled trial
Source: Front Neurosci. 2023 Feb 6;16:1097278. doi: 10.3389/fnins.2022.1097278 (PMC9940791; doi:10.3389/fnins.2022.1097278)
Supplement: Supplementary file 7 [file Table_6.pdf]

frontiers

**Supplementary Table 6.** Gastrointestinal symptoms on Rome-IV Diagnostic Questionnaire.

| Rome-IV items (item number) | Treatment | Odds ratio for greater disturbance (95% CI; *p)** |
|---|---|---|
| *Abdominal pain (40)* | Placebo | Reference |
| | Prebiotic diet | 0.458 (0.135, 1.49; *p* = 0.20) |
| | Probiotic | 1.28 (0.427, 3.86; *p* = 0.66) |
| | Synbiotic | 3.10 (1.00, 9.99; *p* = 0.053) |
| *Hard or lumpy stools (49)* | Placebo | Reference |
| | Prebiotic diet | 1.07 (0.324, 3.52; *p* = 0.92) |
| | Probiotic | 1.87 (0.608, 5.89; *p* = 0.28) |
| | Synbiotic | 1.51 (0.469, 4.96; *p* = 0.49) |
| *Strain during bowel movements (52)* | Placebo | Reference |
| | Prebiotic diet | 0.455 (0.132, 1.49; *p* = 0.20) |
| | Probiotic | 1.27 (4.20, 3.85; *p* = 0.68) |
| | Synbiotic | 2.27 (0.726, 7.25; *p* = 0.16) |
| *Mushy or watery stools (59)* | Placebo | Reference |
| | Prebiotic diet | 1.15 (0.361, 3.67; *p* = 0.81) |
| | Probiotic | 0.761 (0.253, 2.28; *p* = 0.63) |
| | Synbiotic | 0.739 (0.239, 2.27; *p* = 0.60) |
| *Mushy or watery stools following a meal (61)* | Placebo | Reference |
| | Prebiotic diet | 1.11 (0.296, 4.06; *p* = 0.88) |
| | Probiotic | 0.350 (0.074, 1.44; *p* = 0.16) |
| | Synbiotic | 0.513 (0.124, 1.98; *p* = 0.34) |
| *Bloating (65)* | Placebo | Reference |
| | Prebiotic diet | 1.98 (0.630, 6.33; *p* = 0.25) |
| | Probiotic | 0.971 (0.331, 2.84; *p* = 0.96) |
| | Synbiotic | 2.43 (0.821, 7.37; *p* = 0.11) |

*From proportional odds model adjusted for baseline score.
