# Supplementary material for: Effects of a high-prebiotic diet versus probiotic supplements versus synbiotics on adult mental health: The “Gut Feelings” randomised controlled trial
Source: Front Neurosci. 2023 Feb 6;16:1097278. doi: 10.3389/fnins.2022.1097278 (PMC9940791; doi:10.3389/fnins.2022.1097278)

Supplementary Figure 2. Total prebiotic fibre intake at week 20 follow-up, as per purpose-built dietary screener. BL = week 0; W8 = week 8; W20 = week 20.

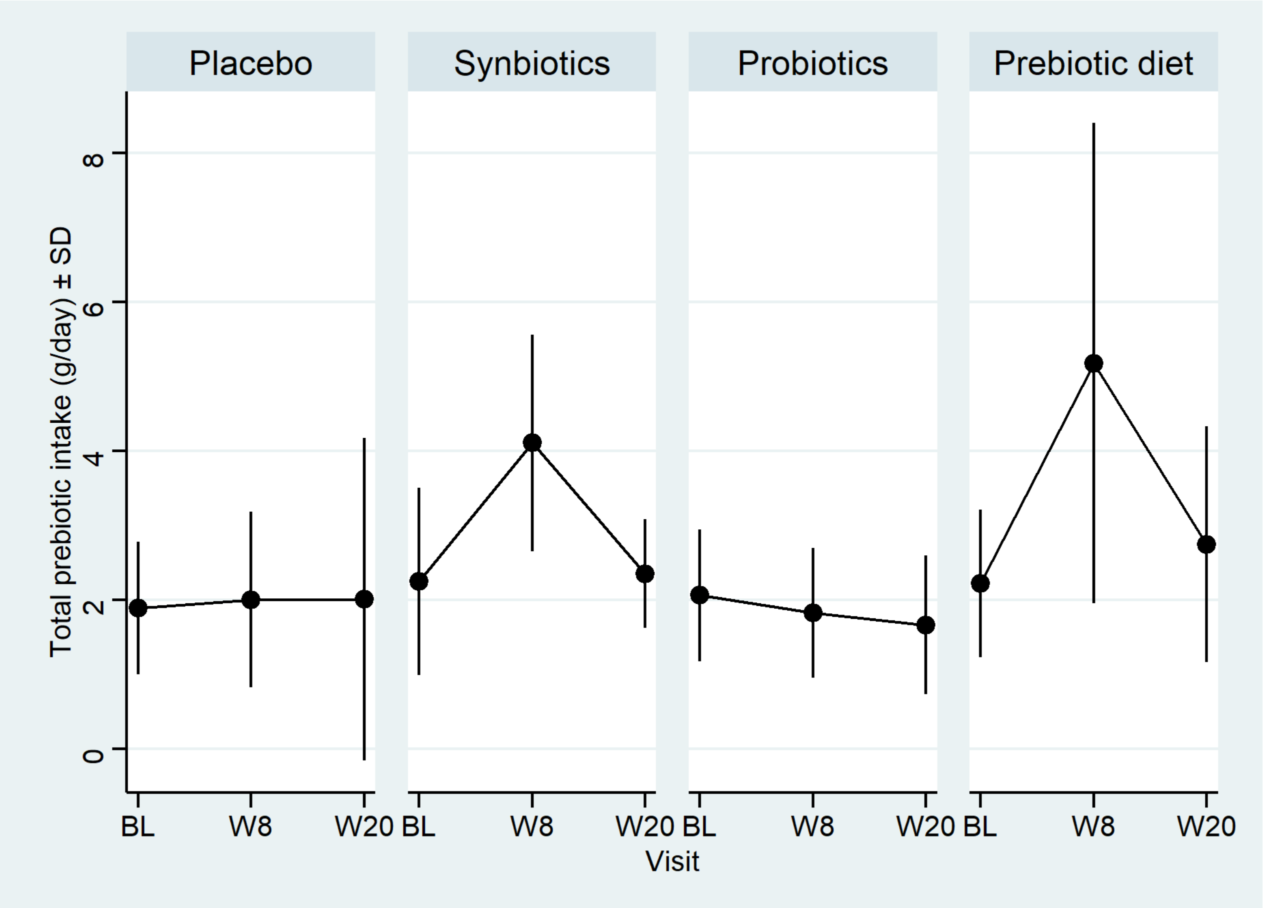

Supplement: Supplementary file 9 [file Image_2.pdf]
