# Supplementary material for: Effects of a high-prebiotic diet versus probiotic supplements versus synbiotics on adult mental health: The “Gut Feelings” randomised controlled trial
Source: Front Neurosci. 2023 Feb 6;16:1097278. doi: 10.3389/fnins.2022.1097278 (PMC9940791; doi:10.3389/fnins.2022.1097278)
Supplement: Supplementary file 10 [file Image_3.pdf]

Supplementary Figure 3. Gastrointestinal symptoms on Rome-IV Diagnostic Questionnaire for **(A)** Item 40, **(B)** Item 49, **(C)** Item 52, **(D)** Item 59, **(E)** Item 61, and **(F)** Item 65.

A

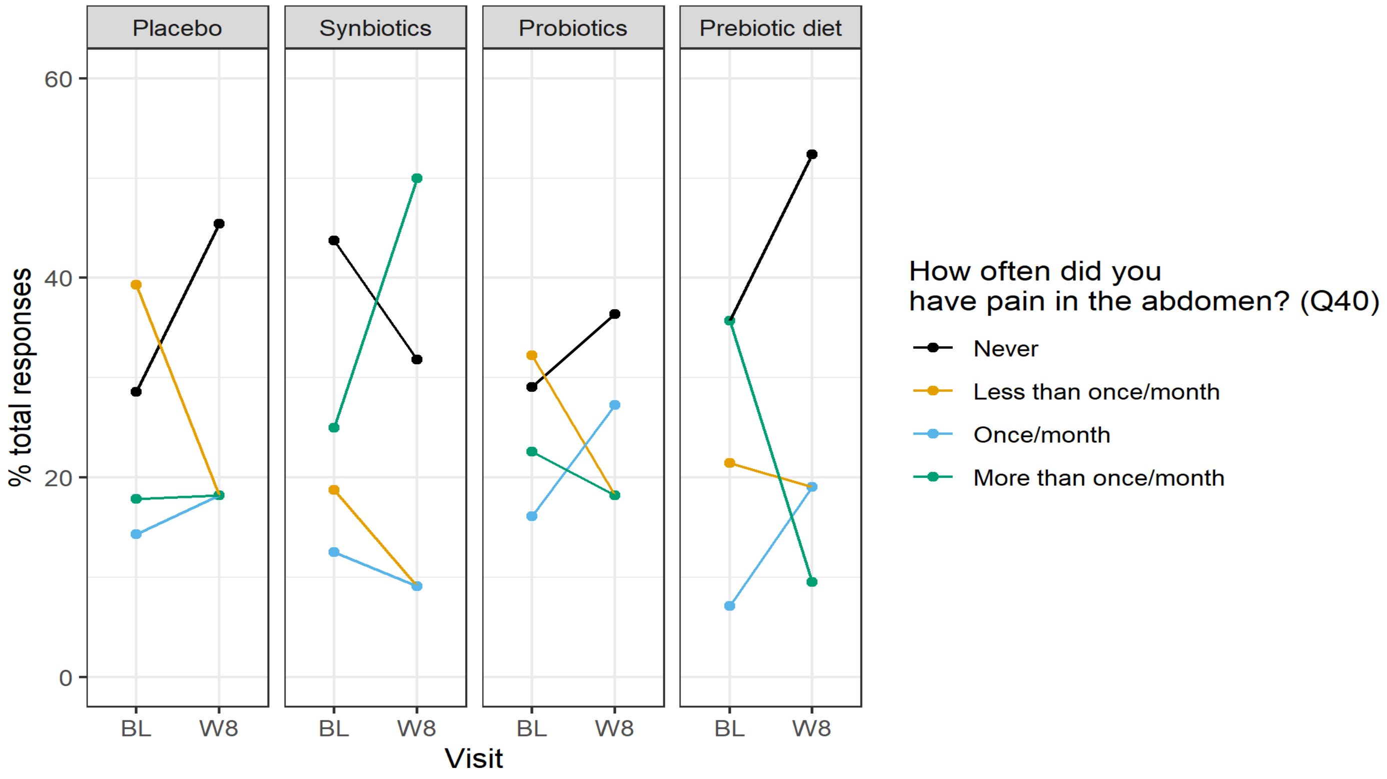

B

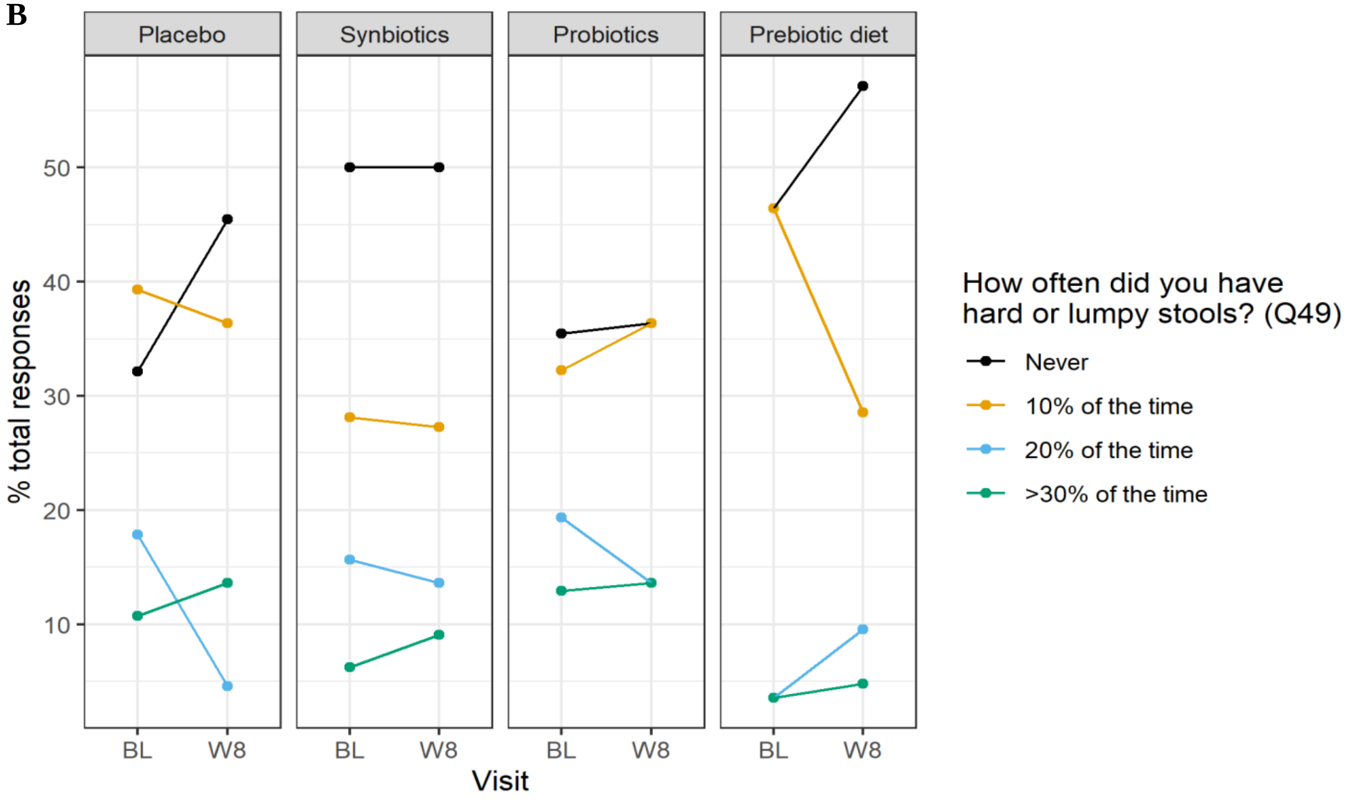

C

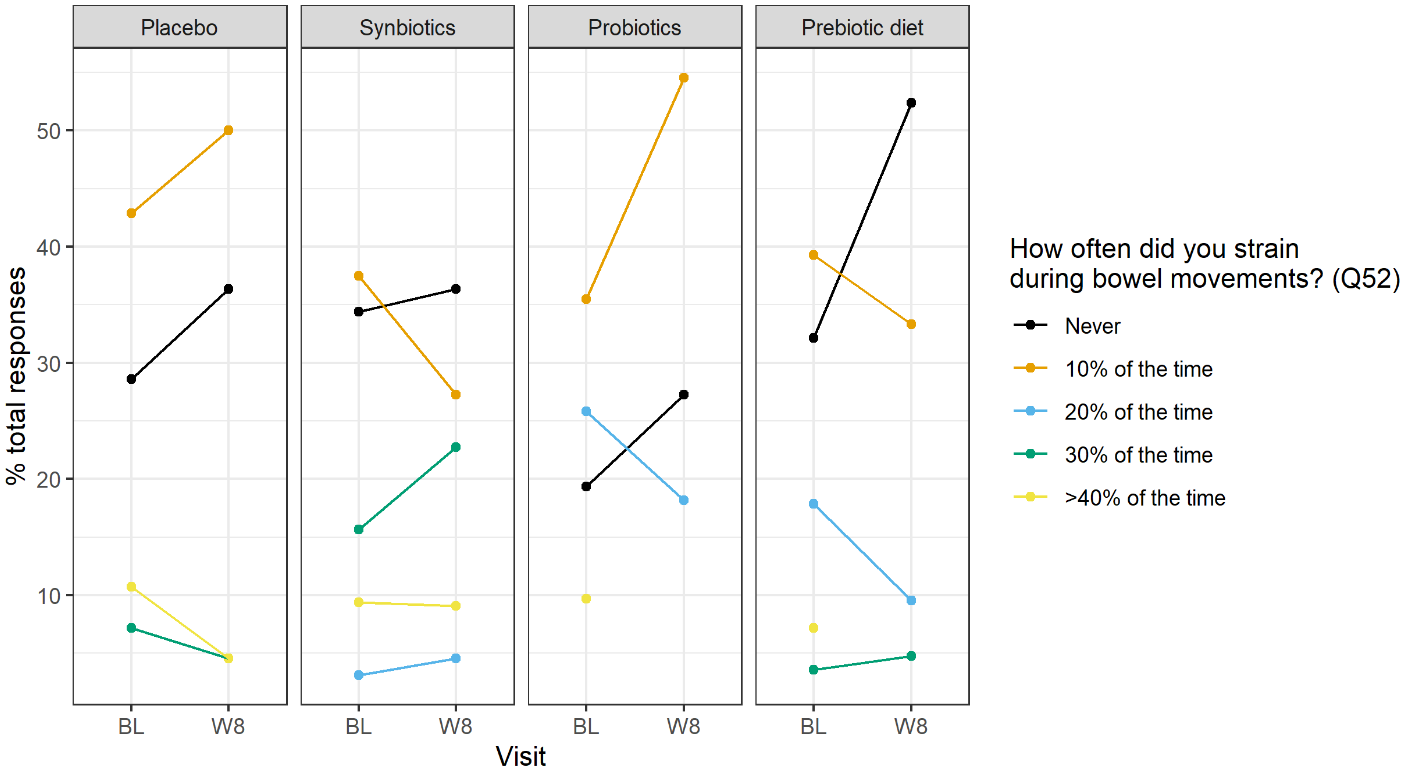
Placebo
Synbiotics
Probiotics
Prebiotic diet
% total responses
10
20
30
40
50
BL
W8
Visit
How often did you strain during bowel movements? (Q52)
Never
10% of the time
20% of the time
30% of the time
>40% of the time

D

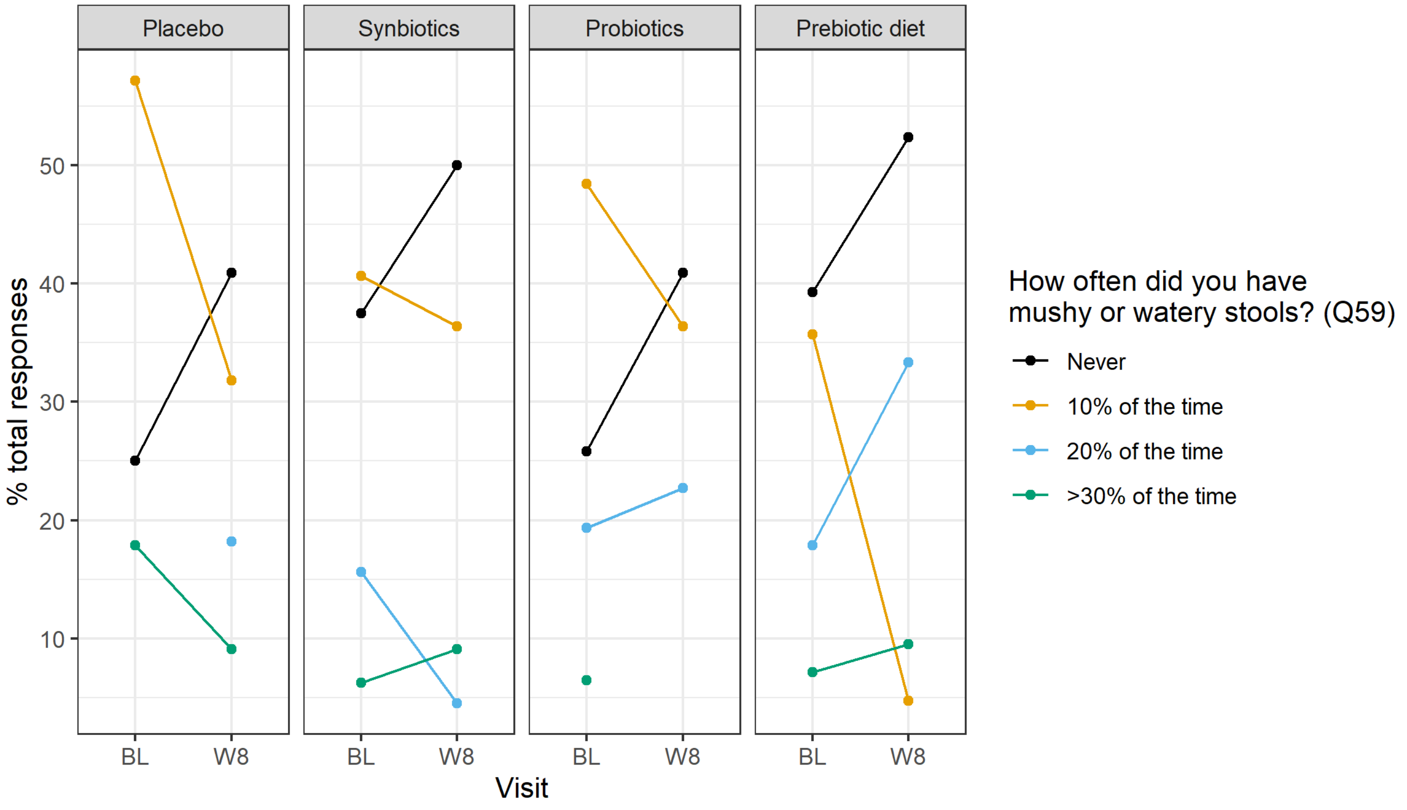
Placebo
Synbiotics
Probiotics
Prebiotic diet
% total responses
10
20
30
40
50
BL
W8
Visit
How often did you have mushy or watery stools? (Q59)
Never
10% of the time
20% of the time
>30% of the time

E

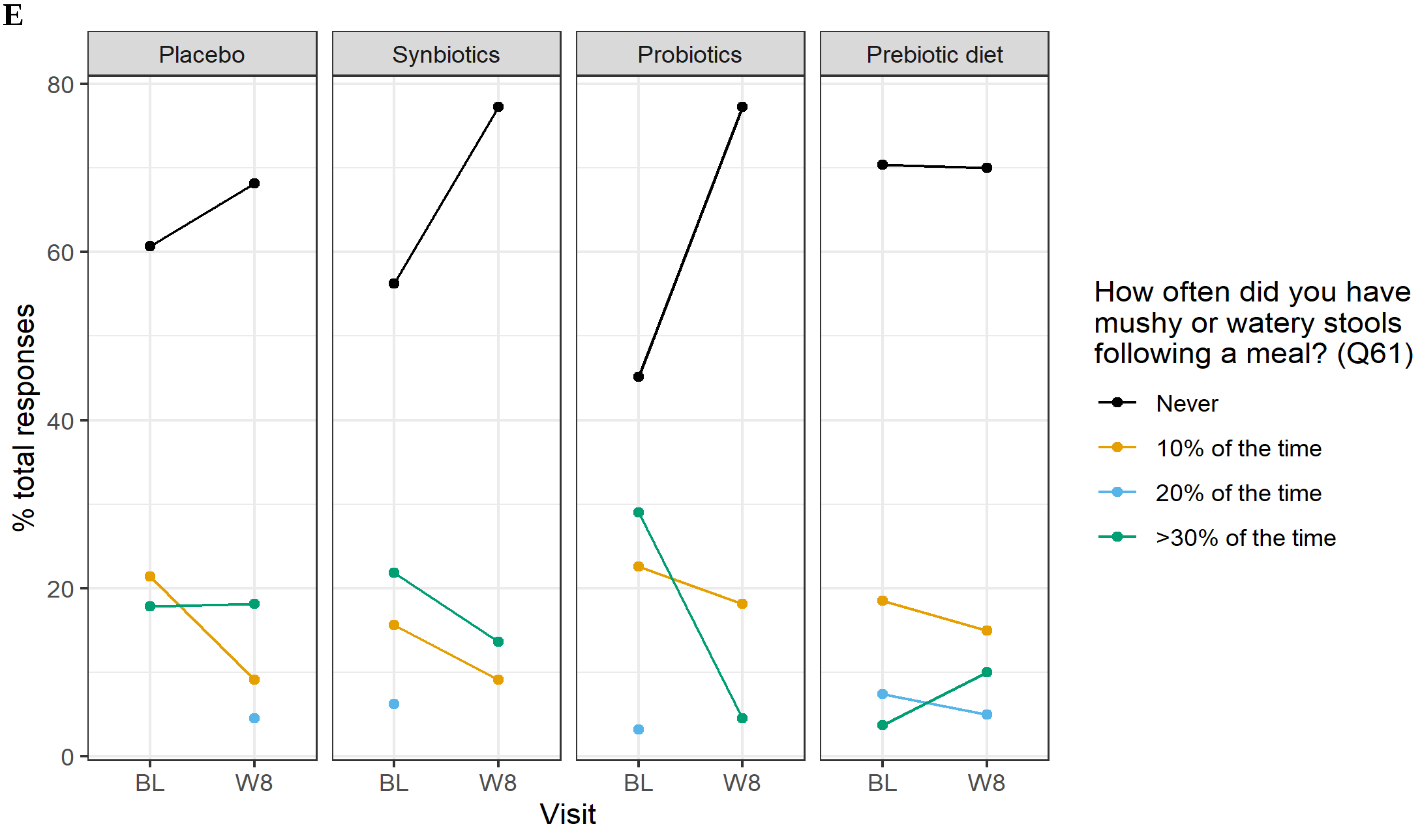

Placebo
Synbiotics
Probiotics
Prebiotic diet
% total responses
0
20
40
60
80
BL
W8
Visit
How often did you have mushy or watery stools following a meal? (Q61)
Never
10% of the time
20% of the time
>30% of the time

F

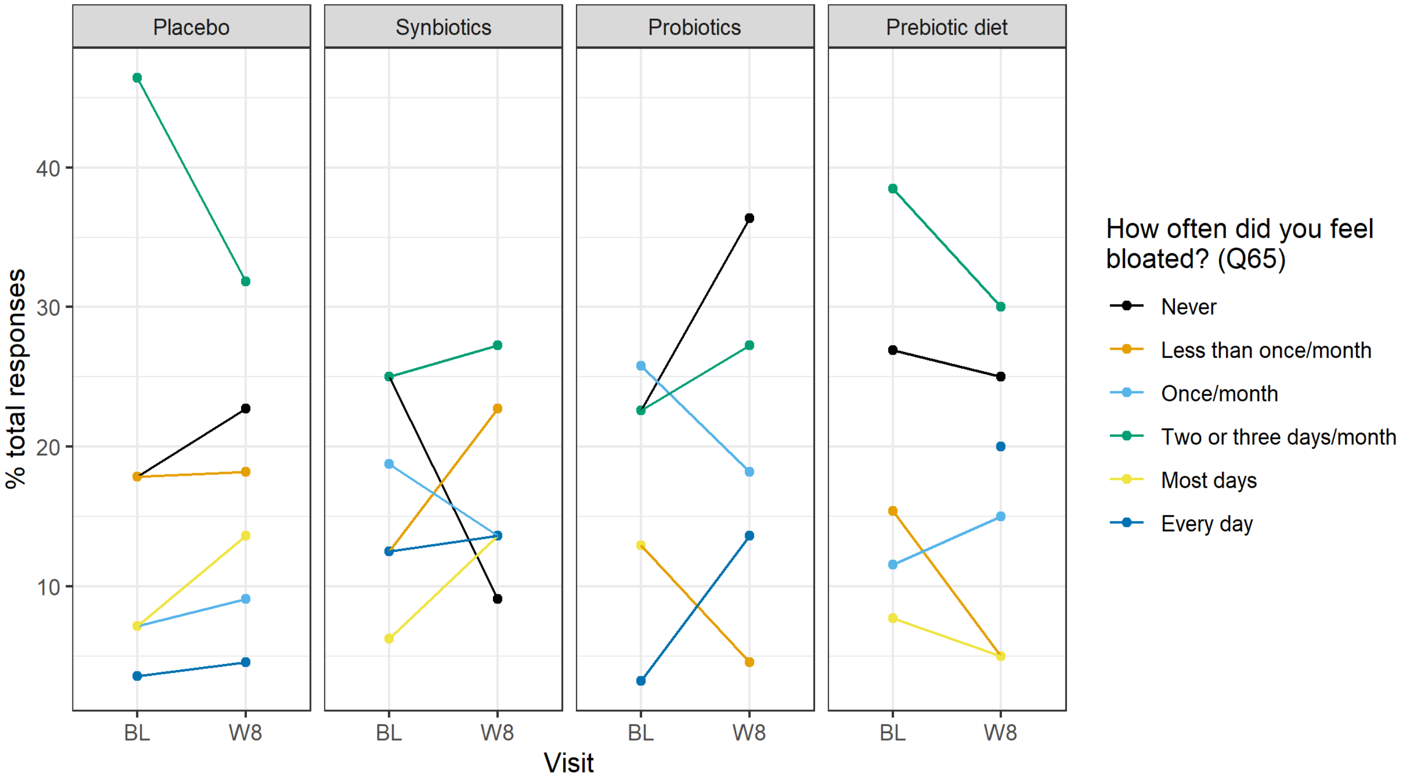

Placebo
Synbiotics
Probiotics
Prebiotic diet
% total responses
10
20
30
40
BL
W8
Visit
How often did you feel bloated? (Q65)
Never
Less than once/month
Once/month
Two or three days/month
Most days
Every day
